# Accurate Measurement of Indoor Radon Concentration using a Low-Effective Volume Radon Monitor

**DOI:** 10.1093/rpd/ncx050

**Published:** 2017-04-17

**Authors:** Aya Tanaka, Nodoka Minami, Yumi Yasuoka, Takeshi Iimoto, Yasutaka Omori, Hiroyuki Nagahama, Jun Muto, Takahiro Mukai

**Affiliations:** ncx0501 Department of Biophysical Chemistry, Kobe Pharmaceutical University, 4-19-1 Motoyamakitamachi, Higashinada-ku, Kobe City, Hyogo 658-8558, Japan; ncx0502 Institute of Radioisotope Research, Kobe Pharmaceutical University, 4-19-1 Motoyamakitamachi, Higashinada-ku, Kobe City, Hyogo 658-8558, Japan; ncx0503 Division for Environment, Health and Safty, The University of Tokyo, 7-3-1 Hongo, Bunkyo-ku, Tokyo 113-8654, Japan; ncx0504 Department of Radiation Physics and Chemistry, Fukushima Medical University, 1 Hikarigaoka, Fukushima City, Fukushima 960-1295, Japan; ncx0505 Department of Earth Science, Tohoku University, 6-3 Aza Aoba, Aramaki, Aoba-ku, Sendai City, Miyagi 980-8578, Japan

## Abstract

AlphaGUARD is a low-effective volume detector and one of the most popular portable radon monitors which is currently available. This study investigated whether AlphaGUARD can accurately measure the variable indoor radon levels. The consistency of the radon-concentration data obtained by AlphaGUARD is evaluated against simultaneous measurements by two other monitors (each ~10 times more sensitive than AlphaGUARD). When accurately measuring radon concentration with AlphaGUARD, we found that the net counts of the AlphaGUARD were required of at least 500 counts, <25% of the relative percent difference. AlphaGUARD can provide accurate measurements of radon concentration for the world average level (~50 Bq m^−3^) and the reference level of workplace (1000 Bq m^−3^), using integrated data over at least 3 h and 10 min, respectively.

## INTRODUCTION

Radon is the cause of thousands of deaths each year in the USA^(1)^. Because it is a radioactive, colorless, odorless and tasteless gas, the measurement of radon levels is the only way to establish the presence of a radon risk^(2)^. As per World Health Organization^(3)^, recommended reference level of indoor radon concentration is 100 Bq m^−3^ while the International Commission on Radiological Protection (ICRP) has recommended reference level of 300 Bq m^−3^ and 1000 Bq m^−3^ for homes and workplaces, respectively^(4)^.

The AlphaGUARD is the most popular portable device with a low-effective volume for radon measurement. Mazed et al. (2007) reported that the time needed to measure 200 Bq m^−3^ with the AlphaGUARD was 15 min^(5)^. Based on that the counts needed to measure with the AlphaGUARD is equivalent to 150 counts. When using only the AlphaGUARD, their criterion of the time needed to measure 200 Bq m^−3^ was the time of the radon activity which has a 10% of relative standard deviation. However, they did not compare the AlphaGUARD with a more sensitive radiation detector.

According to ‘Protocol for Conducting Measurements of Radon and Radon Decay Product in Homes (MAH-2014)’ and ‘Performance Specifications for Instrumentation Systems Designed to Measure Radon Gas in Air (MS-PC-2015)’ of the American Association of Radon Scientists and Technologists, and the American National Standards Institute, the expected range of precision for the individual percent difference is <25% under the condition of radon levels between 74 and 147 Bq m^−3^^(6, 7)^. In Japan, no such protocol has been established. In this study, we analyzed a radon measurement device based on MAH-2014 and MS-PC-2015.

In this study, we investigated the number of counts needed to measure radon concentration with the AlphaGUARD compared with more sensitive radiation detectors. Our analysis parameters were the indoor radon concertation levels (~50 Bq m^−3^) and the highest reference level (1000 Bq m^−3^). We measured radon concentrations of 15–130 Bq m^−3^ and 1000 Bq m^−3^ with three monitors.

## MATERIALS AND METHODS

### Three measuring instruments

Table 1 shows the specifications for three measuring instruments, a gas-flow ionization chamber (DGM-101; Hitachi, Ltd., Japan), a trace environmental level detector with photomultiplier tube (PMT-TEL; Pylon Electronics Inc., Canada), and AlphaGUARD (PQ2000 PRO; Saphymo GmbH, Frankfurt, Germany).

**Table 1.** Specifications of the three measuring instruments and the condition of NIRS radon chamber.

Monitor name	DGM-101^(8)^	PMT-TEL^(10)^	KPU AlphaGUARD^(9)^
**Specifications of monitors**			
Detection limit (Bq m^−3^)	0.64	0.93	2
Detector	Gas-flow ionization chamber	ZnS(Ag) scintillator	Pulse-counting ionization chamber
Effective volume of chamber (L)	14	18	0.56
Gas-flow rate (L min^−1^)	6.5	1.0	No (Diffusion mode)
Data collection interval (min)	1	10	60
**Conditions of NIRS radon chamber**			
Radon level (Bq m^−3^)	1011 ± 72	2066 ± 206	8003 ± 204
Exposure period	94 h	40 h	129 h

The DGM-101 has been used for an airborne radionuclide measurement, needed for the radiation management of a radiation facility^(8)^. The DGM-101 has a large effective volume of 14 L, which contributes to high sensitivity. The DGM-101 operated with a flow rate of 6.5 L min^−1^ and measured the indoor air at 1-min intervals. After passing through a cylindrical miniature glass-fiber filter (P0001071, Saphymo GmbH, Frankfurt, Germany)^(9)^, air was injected into the detector of the DGM-101. The radon concentration was calculated as integrated data per hour or per 10 min.

The PMT-TEL is an atmospheric radon monitor with an effective volume of 18 L^(10)^. It has a thin ZnS (Ag) scintillator with a PMT, and collected radon progenies under electrostatic field inside the chamber. In the present study, it operated with a flow rate of 1.0 L min^−1^ and measured the indoor air at 10-min intervals. The air entered the chamber after passing through a desiccant (Drierite (CaSO_4_); W. A. Hammond Drierite Co. Ltd., USA) and the cylindrical miniature glass-fiber filter. The radon concentration was calculated as integrated data per hour or per 10 min.

The AlphaGUARD is the most popular portable device for radon measurement with an effective volume of 0.56 L^(9, 11–14)^. The AlphaGUARD worked in the diffusion mode and measured the indoor air through the large surface glass-fiber filter (P0002451, Saphymo GmbH, Frankfurt, Germany) at 1-h intervals (the longest interval of the vendor’s specifications) or 10-min intervals. When measuring indoor radon with the AlphaGUARD, the diffusion mode and the 1-h measurement interval are popular setting.

### Conversion into radon concentration

Experiments to obtain conversion/calibration factors of the three measuring instruments were made using a walk-in radon chamber (inner volume: 24.4 m^3^) at the National Institute of Radiological Science, Japan (NIRS radon chamber). The NIRS radon chamber can control radon concentration, temperature and humidity^(15)^. Reference radon concentrations are measured at 10-min intervals by the AlphaGUARD (hereafter NIRS AlphaGUARD) in the diffusion mode which was calibrated at the Physikalisch-Technische Bundesanstalt in Braunschweig, Germany.

Table 1 shows the experimental conditions to obtain the conversion/calibration factors for the DGM-101, the PMT-TEL and AlphaGUARD of Kobe Pharmaceutical University (hereafter KPU AlphaGUARD). For the DGM-101 and the PMT-TEL, the radon concentration was calculated as integrated data per 10 min. By comparing to reference radon concentrations, the conversion factors for the DGM-101 and the PMT-TEL were determined to be 0.56 (fA (Bq m^−3^)^−1^) and 0.61 (cpm (Bq m^−3^)^−1^), respectively. Furthermore, the calibration factor for KPU AlphaGUARD was determined to be 0.99. The background values for the DGM-101, PMT-TEL and KPU AlphaGUARD were measured in radon-free air (N_2_ gas) and determined to be 15 fA, 1.5 cpm, and 8.1 Bq m^−3^, respectively. Based on these results, the measured data (ionization current/count rate/radon concentration) were converted into calibrated radon concentrations using Eqs (1), (2), and (3), respectively:

(1) $$C_{I}=\frac{D_{I}-15}{0.56},$$

(2) $$C_{P}=\frac{D_{P}-1.5}{0.61},$$

(3) $$C_{A}=\frac{D_{A}-8.1}{0.99}.$$

Here, the radon concentrations and measured data were, respectively, *C*_I_ Bq m^−3^ and *D*_I_ fA (DGM-101), *C*_P_ Bq m^−3^ and *D*_P_ cpm (PMT-TEL), and *C*_A_ Bq m^−3^ and *D*_A_ Bq m^−3^ (KPU AlphaGUARD).

The sensitivity of PMT-TEL [0.61 (cpm (Bq m^−3^)^−1^)] in Eq. (2) was higher than the sensitivity of AlphaGUARD [0.05 (cpm (Bq m^−3^)^−1^)]^(9)^. Generally, the sensitivity of detector increases with larger chamber of detector. In this study, the volumes of chamber of the DGM-101 (14 L) and the PMT-TEL (18 L) were larger than the AlphaGUARD (0.56 L) as shown in Table 1.

### Comparing the instruments

Three experiments (Steps 1, 2, and 3) were made for evaluating measurement time and net count required for the indoor radon measurement.

**Figure 1.**
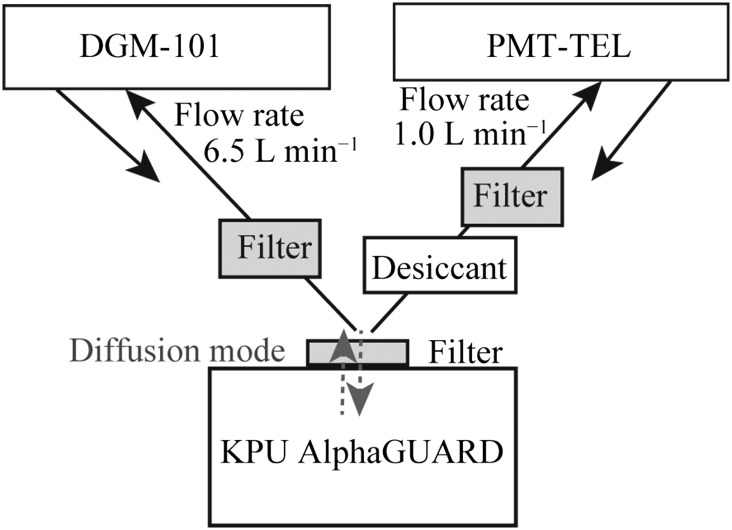
Schematic diagram of three experimental devices.

In Step 1, the indoor radon concentrations were measured simultaneously using the DGM-101 and the PMT-TEL and the KPU AlphaGUARD to examine whether the measuring time of 1 h (Figure 1), a popular setting for AlphaGUARD, is sufficient. If the measuring time of 1 h was not sufficient, we examined the sufficient measuring time would be. These instruments were turned on at least 1 d before the measurements to sufficiently replace the air in the surrounding indoor air.

In Step 2, we examined the response to the range of radon concentrations (15–128 Bq m^−3^), which was wider than the range (26–85 Bq m^−3^) of the Step 1, when comparing DGM-101 and KPU AlphaGUARD. The lower range of the radon concentration was obtained through simultaneous measurements outdoors. In contrast, the higher range was obtained by supplying radon from uranium ore. The KPU AlphaGUARD and uranium ore were placed in a box, while DGM-101 was connected to the box through a tube and the exhaust air from DGM-101 went back into the box. Like Step1, these instruments were started at least 1 d before the measurements. In this experiment, emanation of thoron from the uranium ore into the air was negligibly small by placing the uranium ore into a hermetically sealed plastic bag. It was confirmed through the measurement of thoron concentration in the box by using a radon-thoron discriminative monitor RAD7 (Durridge Company Inc., USA).

In Step 3, the constant high radon concentration (~1000 Bq m^−3^) in the NIRS radon chamber was measured simultaneously using the DGM-101 and the NIRS AlphaGUARD to examine the sufficient measuring time for the highest reference level of indoor radon concentration. The radon concentration measured with the NIRS AlphaGUARD was represented by *C*_AN_ Bq m^−3^. The experiment started at the time when both instruments reached the target radon-concentration level.

## RESULTS

### Radon-concentration levels

**Figure 2.**
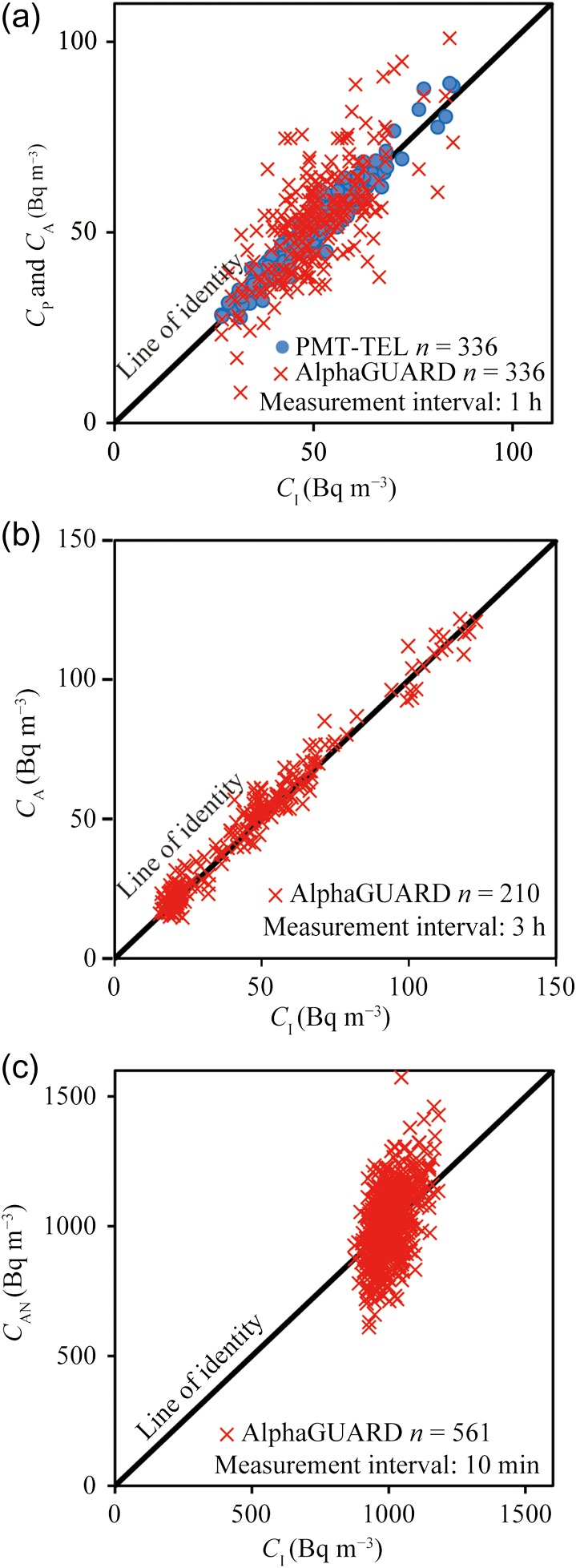
Radon concentrations obtained by the reference monitor (DGM-101) vs. test monitors. The solid black line is the identity line. (**a**) Step 1: the DGM-101 vs. the PMT-TEL and KPU AlphaGUARD. (**b**) Step 2: the DGM-101 vs. the KPU AlphaGUARD. (**c**) Step 3: the DGM-101 vs. the NIRS AlphaGUARD.

In Step 1, Figure 2a shows the relationship between the *C*_I_ group and the *C*_P_ and *C*_A_ groups based on the hourly data. The *C*_A_ results suggest considerable variability compared to the *C*_P_ results. The question of whether the *C*_P_ (or *C*_A_) group can be considered consistent with the *C*_I_ group is addressed in the next section. The average radon concentration ($\overset{¯}{C_{I}}$) of the *C*_I_ group was 52.3 Bq m^−3^ within the range 26–85 Bq m^−3^. This radon level was close to the worldwide arithmetic median values of dwellings, that is, 46 Bq m^−3^^(16)^.

In Step 2, Figure 2b shows the relationship between the *C*_I_ group and the *C*_A_ groups based on the 3-h data for the narrowing of the width of the required net counting range obtained in Step 1. The average radon concentration ($\overset{¯}{C_{I}}$) of the *C*_I_ group was 45 Bq m^−3^ within the range 15–128 Bq m^−3^.

In Step 3, Figure 2c shows the relationship between the *C*_I_ group and the *C*_AN_ groups based on the 10-min data. The average radon concentration ($\overset{¯}{C_{I}}$) of the *C*_I_ group was 1003 Bq m^−3^ within the range 875–1182 Bq m^−3^.

## DISCUSSION

### Criteria

**Figure 3.**
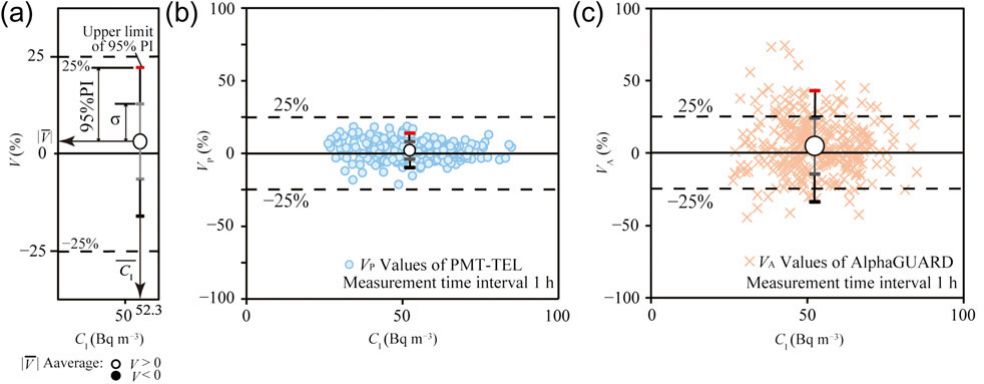
Evaluation of the range of the 95% PI of $\overset{¯}{V}$% using integrated hourly data (*n* = 336). The relationship between the relative percent difference *V*% and the reference radon concentration (*C*_I_ Bq m^−3^). (**a**) Explanation of symbols in Figures 3b and c, 4, 5 and 6; (**b**) The range of the 95% PI_p_ of ${\overset{¯}{V}}_{P}$; (**c**) The range of the 95% PI_A_ of ${\overset{¯}{V}}_{A}$.

The consistencies of the values measured using the PMT-TEL and the AlphaGUARD were evaluated based on the radon concentrations measured by the DGM-101. The relative percent difference, *V*_P_% (*V*% in Figure 3a), was given by

(4) $$V_{P}=\frac{100(C_{P}-C_{I})}{C_{I}}.$$

*V*_P_% is the degree by which a single measurement value *C*_P_ Bq m^−3^ deviated from the reference values (*C*_I_ Bq m^−3^). The *C*_P_ and *C*_I_ Bq m^−3^ measured simultaneously were used.

Similarly, the relative percent difference, *V*_A_% was given by

(5) $$V_{A}=\frac{100(C_{A}-C_{I})}{C_{I}}.$$

*V*_A_% is the degree by which a single measurement value *C*_A_ (or *C*_AN_) Bq m^−3^ deviates from the reference values (*C*_I_ Bq m^−3^). The *C*_A_ (or *C*_AN_) and *C*_I_ Bq m^−3^ measured simultaneously were used.

The average value and standard deviation $\overset{¯}{V}\pm \sigma$ % [(${\overset{¯}{V}}_{P}\pm \sigma_{P}$) or (${\overset{¯}{V}}_{A}\pm \sigma_{A}$)] was calculated for each group. The ranges of the 95% PI were also calculated and the upper limit of 95% PI_P_ and the upper limit of 95% PI_A_ were given by Eqs (6) and (7), respectively:

(6) $$\left|{\overset{¯}{V}}_{P}\right|+k\sigma_{P}\sqrt{\left(1+\frac{1}{n}\right)}=\left|{\overset{¯}{V}}_{P}\right|+\gamma \sigma_{P},$$

(7) $$\left|{\overset{¯}{V}}_{A}\right|+k\sigma_{A}\sqrt{\left(1+\frac{1}{n}\right)}=\left|{\overset{¯}{V}}_{A}\right|+\gamma \sigma_{A}.$$

**Figure 4.**
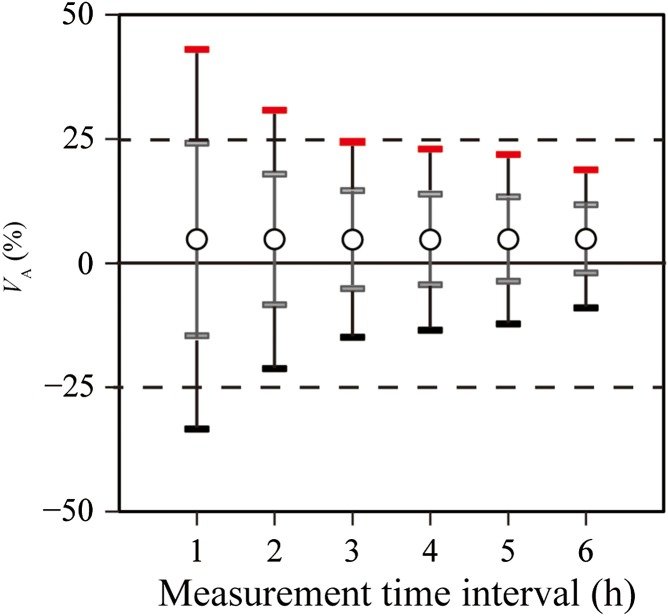
Evaluation of the range of the 95% PI_A_ of ${\overset{¯}{V}}_{A}$ using integrated hourly data obtained by the KPU AlphaGUARD.

**Figure 5.**
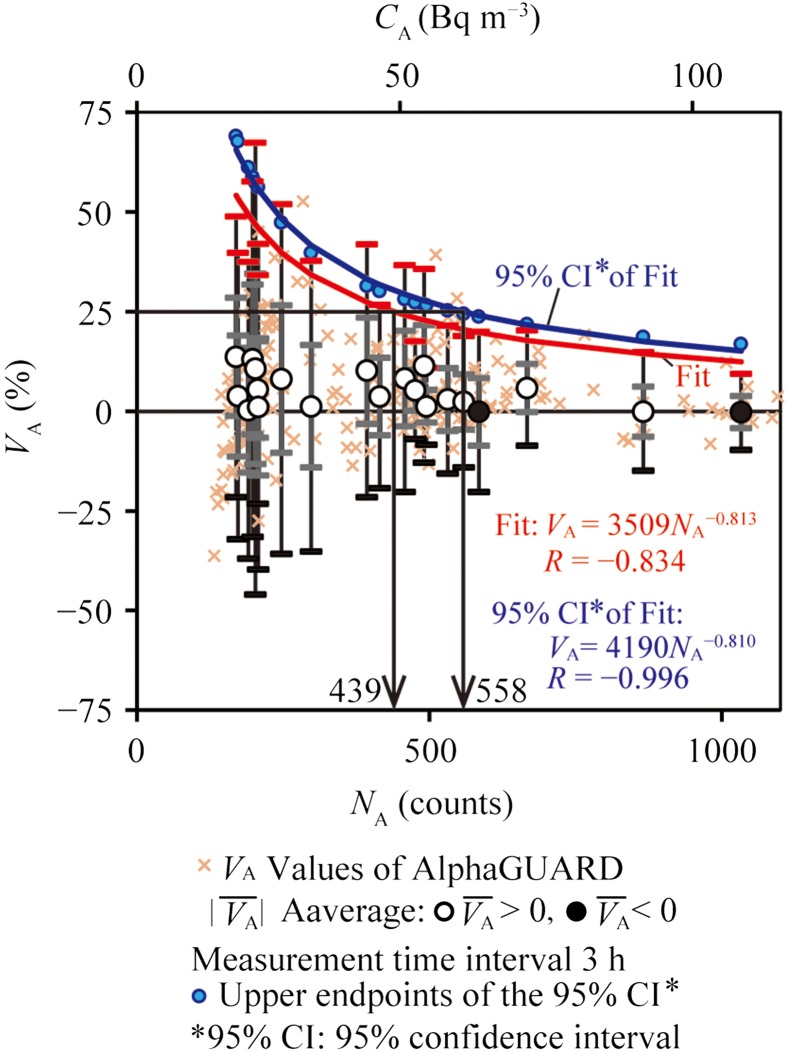
Evaluation of the range of the 95% PI_A_ of ${\overset{¯}{V}}_{A}$ using the *N*_A_ obtained by the KPU AlphaGUARD. Radon concentration: ~50 Bq m^−3^.

Here, the value of *k* indicates the student’s *t*-value at 0.05 significance for a 2-tailed *t*-test, which was determined based on the degree of freedom (*n*− 1). We determined $\left|{\overset{¯}{V}}_{A}\right|$, which is the absolute value of ${\overset{¯}{V}}_{A}$. In Figures 3–5, the open circles were ${\overset{¯}{V}}_{A}$ > 0 and the closed circles were ${\overset{¯}{V}}_{A}$ < 0. When the $\left|{\overset{¯}{V}}_{P}\right|+\gamma \sigma_{P}$ (or $\left|{\overset{¯}{V}}_{A}\right|+\gamma \sigma_{A}$) was <25%, the individual percent difference was used to meet the efficiency criteria. The outlines in the case of *V* > 0 are depicted in Figure 3a.

### PMT-TEL

In Step 1, the *C*_I_ and *V*_P_ values were calculated based on integrated hourly data (number of data points: *n* = 336; *γ* = 1.97). Figure 3b shows that the $\left|{\overset{¯}{V}}_{P}\right|+\gamma \sigma_{P}$ was within 25% and ${\overset{¯}{V}}_{P}$ was 2.0%. Therefore, the *C*_P_ group was considered to be consistent with the *C*_I_ group. PMT-TEL and DGM-101 on integrated hourly data were enough to measure indoor radon concentration.

### AlphaGUARD

In Step 1, the *C*_I_ and *V*_A_values were calculated based on integrated hourly data (*n* = 336; *γ* = 1.97). Although ${\overset{¯}{V}}_{A}$ was 4.8%, the $\left|{\overset{¯}{V}}_{A}\right|+\gamma \sigma_{A}$ was not within 25%, as shown in Figure 3c. Secondly, to determine the appropriate measurement time interval, the *C*_I_ and *V*_A_ values were calculated on the basis of data integrated over 2-h (*n* = 168; *γ* = 1.98), 3-h (*n* = 112; *γ* = 1.99), 4-h (*n* = 84; *γ* = 2.00), 5-h (*n* = 67; *γ* = 2.01) or 6-h (*n* = 56; *γ* = 2.02) intervals. The *C*_I_ and *V*_A_ groups were evaluated using the same method as above. Figure 4 shows that the $\left|{\overset{¯}{V}}_{A}\right|+\gamma \sigma_{A}$ calculated based on data integrated over an interval between 2 and 4 h (~3 h) was within 25% in the average radon concentration 52.3 Bq m^−3^. We considered that the net counts (*N*_A_), which the KPU AlphaGUARD was required, were within the range between 314 and 628 (~471) counts. The *N*_A_ was the net counts of the conversion result from radon concentration using the sensitivity of 0.05 cpm (Bq m^−3^)^−1^.

In Step 2, the *C*_I_ values, *V*_A_values and *N*_A_ values of the KPU AlphaGUARD, which were calculated based on the integrated 3-h data, were listed in increasing order of *C*_I_. ${\overset{¯}{V}}_{A}\pm \sigma_{A}$ and ${\overset{¯}{N}}_{A}\pm \sigma_{NA}$ were the averages and standard deviation, which averaged every 10 data values (*n* = 10) of *V*_A_ and *N*_A_, respectively. Figure 5 shows the relationship with ${\overset{¯}{N}}_{A}$ and $\left|{\overset{¯}{V}}_{A}\right|$ (or $\left|{\overset{¯}{V}}_{A}\right|\pm \sigma_{A}$ or $\left|{\overset{¯}{V}}_{A}\right|\pm \gamma \sigma_{A}$). In Figure 5, a power-law regression curve (the red curve represented Fit in Figure 5) and the upper limits of the 95% confidence interval (95% CI; blue filled circles in Figure 5) was made using the values of $\left|{\overset{¯}{V}}_{A}\right|+\gamma \sigma_{A}$ (red error bars in Figure 5). Moreover, a power-law regression curve was drawn for an upper limit of the 95% CI of Fit. We found *N*_A_ of the KPU AlphaGUARD 439 and 558 counts (on the *X*-axis) corresponding to the 25% of *V*_A_ (on the *Y*-axis) on the Fit and 95% CI of Fit in Figure 5, respectively. When the KPU AlphaGUARD could measure variable indoor radon levels accurately, we considered that ~500 counts were required in Step 2.

**Figure 6.**
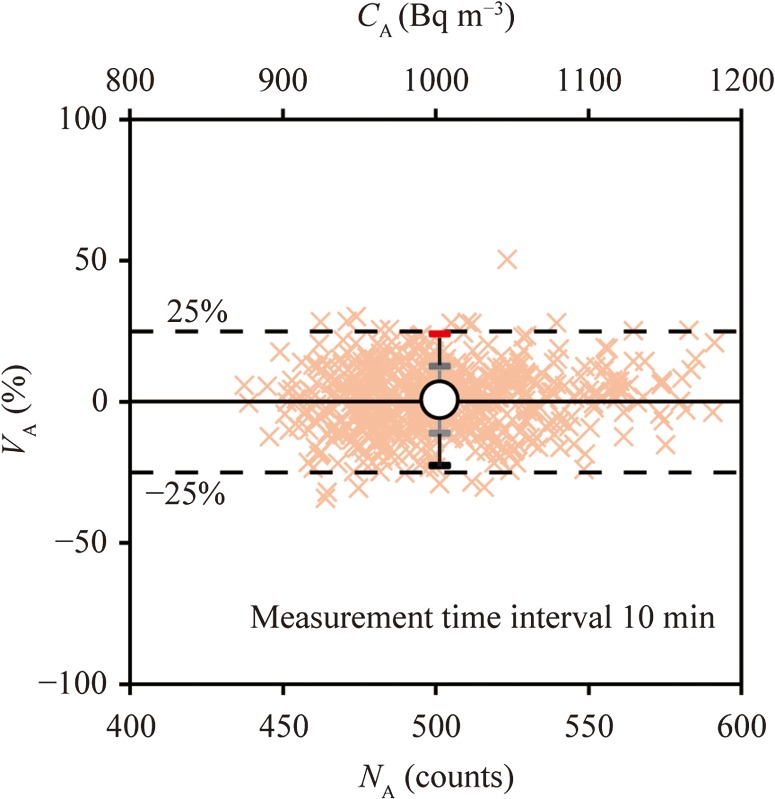
Evaluation of the range of the 95% PI of $\bar{V}$_A_% using *N*_A_ obtained by the NIRS AlphaGUARD. Radon concentration: ~1000 Bq m^−3^.

In Step 3, when the NIRS AlphaGUARD measures radon concentrations of 1000 Bq m^−3^ at 10-min intervals, the net counts are estimated at ~500 counts. When the radon concentration was kept at 1000 Bq m^−3^ at the NIRS radon chamber, the radon concentration was simultaneously measured with the NIRS AlphaGUARD (diffusion mode) and the DGM-101. Figure 2c shows the relationship between the *C*_I_ group and the *C*_AN_ groups based on 10-min intervals data. The *C*_I_ and *V*_A_ values were calculated based on integrated 10-min intervals data (*n* = 561; *γ* = 1.97). The $\left|{\overset{¯}{V}}_{A}\right|+\gamma \sigma_{A}$ was 24% within 25%, as shown in Figure 6. The average counts of the NIRS AlphaGUARD were 505 counts with the sensitivity of NIRS AlphaGUARD being 0.05 cpm (Bq m^−3^)^−1^. In Step 3, we considered that ~500 counts were required as the *N*_A_ of the NIRS AlphaGUARD.

When the AlphaGUARD could measure radon concentration accurately, we considered that *N*_A_ (the net counts of the AlphaGUARD) were required of at least 500 counts, which was greater than the published data (150 counts).

## CONCLUSIONS

The consistency of indoor radon-concentration data was evaluated based on simultaneous measurements by a DGM-101, a PMT-TEL, and AlphaGUARD. The DGM-101 and the PMT-TEL were ~10 times more sensitive than AlphaGUARD. It was established that the DGM-101 and the PMT-TEL could provide accurate radon-concentration data by sampling at an hourly rate of indoor radon levels. When accurately measuring radon concentration with AlphaGUARD, we found that the net counts of the AlphaGUARD were at least 500 counts, <25% of the relative percent difference. The result of this study suggests that AlphaGUARD can provide accurate measurements of radon concentration for the world average level (~50 Bq m^−3^) and the reference level of workplace (1000 Bq m^−3^), using integrated data over at least 3 h and 10 min, respectively.

## FUNDING

This study was supported by the Ministry of Education, Culture, Sports, Science and Technology (MEXT) of Japan, under its Earthquake and Volcano Hazards Observation and Research Program, and by Tokio Marine Kagami Memorial Foundation.

## References

[1] National Research Council (U.S. NRC) Committee on Health Risks of Exposure to Radon. Health Effects of Exposure to Radon (Vol. VI) (Washington, D.C.: National Academies Press) (1999).

[2] United States Environmental Protection Agency (U.S.EPA) A Citizen’s Guide to Radon. Available on https://www.epa.gov/radon/publications-about-radon (22 February 2017, data last accessed).

[3] World Health Organization (WHO) WHO Handbook on Indoor Radon: a Public Health Perspective (Geneva: WHO) (2009). 23762967

[4] ICRP 115 (International Commission on Radiological Protection) Lung cancer risk from radon and progeny and statement on radon. ICRP Publication 115 Ann. ICRP 40 (2010). 10.1016/j.icrp.2011.08.011 22108246

[5] Mazed D., Ciolini R., Curzio G. and Del Gratta A. A new active method for continuous radon measurements based on a multiple cell proportional counter. Nucl. Instrum. Methods Phys. Res. Sect. A 582, 535–545 (2007).

[6] The American Association of Radon Scientists and Technologists (AARST) and the American National Standards Institute (ANSI) Protocol for Conducting Measurements of Radon and Radon Decay Product in Homes (MAH-2014) (North Carolina: AARST) (2014).

[7] The American Association of Radon Scientists and Technologists (AARST) and the American National Standards Institute (ANSI) Performance Specifications for Instrumentation Systems Designed to Measure Radon Gas in Air (MS-PC-2015) (North Carolina: AARST) (2015).

[8] Hitachi, Ltd User Manual of Gas-flow Ionization Chamber (Tokyo: Hitachi, Ltd.) (1990) (in Japanese).

[9] Saphymo GmbH Portable Radon Monitor, User Manual (2012). Available on http://www.saphymo.com/radiation-measurement/environmental-radiation-monitoring-systems/alphaguard/154.htm (28 March 2017, data last accessed).

[10] Pylon Electronics Inc Trace Level Radon Gas Detector Instruction Manual (Ottawa: Pylon Electronics Inc.) (2001).

[11] Ramola R. C., Kandari M. S., Negi M. S. and Choubey V. M. A study of diurnal variation of indoor radon concentrations. Jpn. J. Health Phys. 35, 211–216 (2000).

[12] Yasuoka Y., Ishikawa T., Tokonami S., Takahashi H., Sorimachi A. and Shinogi M. Radon mitigation using an air cleaner. J. Radioanal. Nucl. Chem. 279, 885–891 (2009).

[13] Papachristodoulou C. A., Patiris D. L. and Ioannides K. G. Exposure to indoor radon and natural gamma radiation in public workplaces in north-western Greece. Radiat. Meas. 45, 865–871 (2010).

[14] Mostafa A. M. A., Yamazawa H., Uosif M. A. M. and Moriizumi J. Seasonal behavior of radon decay products in indoor air and resulting radiation dose to human respiratory tract. J. Radiat. Res. Appl. Sci. 8, 142–147 (2015).

[15] Janik M., Tokonami S., Kovács T., Kávási N., Kranrod C., Sorimachi A., Takahashi H., Miyahara N. and Ishikawa T. International intercomparisons of integrating radon detectors in the NIRS radon chamber. Appl. Radiat. Isot. 67, 1691–1696 (2009). 19375331 10.1016/j.apradiso.2009.03.006

[16] United Nations Scientific Committee on the Effects of Atomic Radiation (UNSCEAR) Sources and Effects of Ionizing Radiation. UNSCEAR 2000 Report to the General Assembly, with Scientific Annexes, Volume I (New York: United Nations Publications) (2000).

